# Immobilization of the Wrist After Dorsal Wrist Ganglion Excision: A Systematic Review and Survey of Current Practice

**DOI:** 10.1177/15589447211014631

**Published:** 2021-06-06

**Authors:** Chloe R. Wong, Marta Karpinski, Alexandra C. Hatchell, Mark H. McRae, Jessica Murphy, Matthew C. McRae

**Affiliations:** 1 McMaster University, Hamilton, ON, Canada; 2 St. Joseph’s Healthcare Hamilton, ON, Canada

**Keywords:** wrist, anatomy, soft tissue reconstruction, trauma, diagnosis, surgery, specialty, treatment, research and health outcomes, outcomes

## Abstract

**Background:**

Postoperative care after dorsal wrist ganglion (DWG) excision is highly varied. The effect of immobilization of the wrist on patient outcomes has not yet been examined.

**Methods:**

A systematic review of the literature was performed to determine whether wrist immobilization after DWG surgical excision is beneficial. A survey of hand surgeons in Canada was performed to sample existing practice variations in current immobilization protocols after DWG excision.

**Results:**

A systematic review yielded 11 studies that rigidly immobilized the wrist (n = 5 open excision, n = 5 arthroscopic excision, n = 1 open or arthroscopic excision), 10 studies that used dressings to partially limit wrist motion (n = 5 open, n = 5 arthroscopic), 1 study (open) that did either of the above, and 2 studies (arthroscopic) that did not restrict wrist motion postoperatively. This ranged from 48 hours to 2 weeks in open DWG excision and 5 days to 3 weeks in arthroscopic DWG excision. The survey of Canadian hand surgeons had a similarly divided result of those who chose to immobilize the wrist fully (41%), partially (14%), or not at all (55%). Most surgeons surveyed who immobilized the wrist postoperatively did so for 1 to 2 weeks.

**Conclusion:**

The systematic review and survey of Canadian hand surgeons reveal that hand surgeons are divided regarding the need to immobilize the wrist after DWG excision. In terms of functional outcome, there is no compelling data to suggest 1 strategy is superior. The time frame for immobilization when undertaken was short at 2 weeks or less.

The systematic review is registered in the PROSPERO database (PROSPERO 2016:CRD42016050877).

## Introduction

Ganglions are the most common tumor of the hand and are commonly found on the dorsal aspect of the wrist, arising from the wrist capsule at the scaphoid-lunate interval.^1^ The cause of dorsal wrist ganglion (DWG) remains unknown.^2-4^ While typically firm, round, and painless, compression of nearby terminal branches of the posterior interosseous nerve may cause pain.^1,5,6^ Treatment options include no intervention, needle aspiration, and surgical excision. Recurrence rates vary from 25% to 67% for patients treated with needle aspiration and between 8.3% and 42% after surgical excision.^7-9^

Despite the prevalence of DWG and routine surgical treatment, there is a lack of consensus among hand surgeons as to the length of time a wrist should be immobilized postoperatively, if at all. The objectives of this study were to conduct a systematic review of the literature to examine outcomes following various immobilization protocols and survey Canadian hand surgeons to assess the variability in postoperative protocols following DWG excision.

## Methods

### Systematic Review

#### Search strategy and study selection

This review was reported in accordance with the Preferred Reporting Items for Systematic Reviews and Meta-Analyses (PRISMA) guidelines. This systematic review has been registered in the PROSPERO database (PROSPERO 2016:CRD42016050877). The following databases were searched: CINAHL, Embase, MEDLINE, and SportDiscus on September 13, 2016, and repeated on January 3, 2020 (refer to Supplemental Material for search strategy).

Included, full-text articles were: (1) a randomized controlled trial (RCT) comparing different postoperative splinting protocols in DWG excision patients; or (2) any other RCT, observational study, or case series specifying a postoperative care protocol for DWG excision patients. Articles were excluded if they demonstrated any of the following: an entirely pediatric population, nonsurgically managed DWG, case studies, and non-English articles. All outcomes were considered, including grip strength, range of motion, pain, quality of life, and recurrence rates.

#### Data abstraction and collection

Studies were screened by 3 independent reviewers (A.C.H., M.K., and C.R.W.). Reviewers conducted a pilot screen of the titles and abstracts of the first 50 articles to practice applying the inclusion/exclusion criteria. Once consistency was established, title and abstract screening was completed and authors proceeded with full-text review of the identified studies. In the case of disagreement, the senior author (M.C.M.) was consulted. Interrater agreement for both review phases was calculated using an unweighted kappa statistic. Data were extracted using a predefined, standardized data collection instrument. Extracted data included demographic information, the type of surgical intervention, the postoperative protocol, follow-up duration, and any reported outcomes. The updated review was performed by 2 independent reviewers (C.R.W. and M.H.M.).

#### Risk of bias assessment

Risk of bias (RoB) was assessed individually by 2 reviewers (C.R.W. and M.H.M.). Disagreements were resolved through discussion until consensus was achieved, and consultation with an arbitrator (M.C.M.), if needed. Included studies that were nonrandomized were evaluated using the Risk of Bias in Non-Randomized Studies-of Interventions (ROBINS-I) scale, and randomized studies were evaluated using the Cochrane RoB 2.0 tool.^10,11^

### Survey of Hand Surgeons

Following the systematic review of the literature, a survey was conducted to assess current postoperative dressings and immobilization protocols by hand surgeons in Canada.

The survey was first piloted in a small sample of McMaster University senior plastic surgery residents and staff to gather feedback. Using the Canadian Society of Plastic Surgeons 2016 Member Roster, all Canadian plastic surgeons who perform hand surgery were mailed a copy of the survey, a personalized cover letter explaining the purpose of the survey, and a return envelope. This study was exempt from ethics review by a research ethics officer. All procedures followed were in accordance with the ethical standards of the responsible committee on human experimentation (institutional and national) and with the Helsinki Declaration of 1975, as revised in 2008. Informed consent was obtained from all participants (surgeons) being included in the survey.

### Data Analysis

All data from the systematic review and survey were summarized descriptively. Proportions and descriptive statistics were reported, where applicable.

## Results

### Systematic Review

#### Article selection

**Figure 1.**
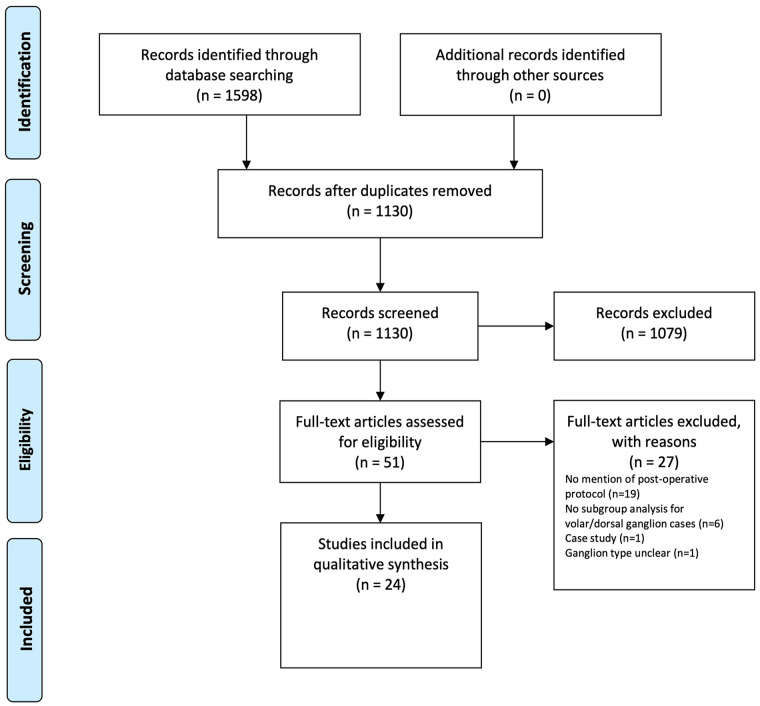
Preferred Reporting Items for Systematic Reviews and Meta-Analyses diagram.

The database search identified 1598 articles, 51 of which underwent full-text review. Of these, 24 articles met inclusion criteria and were included in the review (see PRISMA flow diagram presented in Figure 1). Agreement between reviewers in the first review stage was substantial (kappa = 0.76), and almost perfect in the second stage (kappa = 0.81).

#### Characteristics of included studies

Of the articles included, 3 were RCTs and 21 were observational studies. Eleven studies involved open DWG excision (Table 1), 12 studies involved arthroscopic DWG excision (Table 2), and 1 study involved both open and arthroscopic DWG excision (Tables 1 and 2).

**Table 1.** Summary of Studies (Open Dorsal Wrist Ganglion Excision).

Study	Population	Postoperative protocol	Postoperative outcomes
Angelides and Wallace^12^	500 patients (380 females, 120 males); 454 primary ganglions, 46 recurrent ganglions; average age: 29.6 y (range: 4-71)	Bulky bandage or plaster splint applied (length of time not specified)	*Average follow-up duration*: 9 mo. *Recurrence rate*: 0.9% *ROM*: 1.2% experienced loss of 0° to 10° volar flexion
Balazs et al^13^	125 active duty military personnel (40 females, 85 males); average age: 31 (range: 18-51)	Volar orthosis applied for 2 wk; no push-ups or heavy lifting until 12 wk postoperatively	*Average follow-up duration*: 45 mo. *Recurrence rate*: 9% 15% granted permanent push-up waiver (due to pain or stiffness)
Clay and Clement^14^	61 patients (42 females, 19 males); average age: 32 y (range: 13-59)	Plaster slab applied for 10 d, after which active mobilization was encouraged	*Average follow-up duration*: 28 mo; 51 patients available for follow-up *Recurrence rate*: 6% *Pain*: 79% reported an improvement in pain/discomfort *Grip strength*:18% complained of weakened grip postoperatively *ROM*: normal, 8% reported discomfort at flexion/extension extremes
Gündeş et al^15^	24 patients with DWG (17 females, 7 males); average age: 30.2 y (range: 16-52)	Bulky compressive dressing applied for 2 wk	*Average follow-up duration*: 27 mo *Recurrence rate*: 8.3% *Pain*: 11.5% reported wrist pain postoperatively *ROM*: 11.5% had a 20° or more decrease in ROM
Hwang et al^16^	18 patients (17 females, 1 male); average age: 28.4 y (range: 9-50)	Bulky compressive dressing applied to hold wrist in 20° extension; active ROM exercises started after 2 wk	*Average follow-up duration*: 12.7 mo *Recurrence rate*: 5% *ROM*: 11° decrease in flexion after surgery (*P* < .01)
Jagers Op Akkerhuis et al^17^	25 patients receiving surgical excision for DWG	Pressure bandage applied for 48 h postoperatively	*Recurrence rate*: 28%
Janzon and Niechajev^18^	127 patients with DWG (81 females, 46 males)	Immobilization ranged from 7 to 10 d	*Average follow-up duration*: 5 y; 111 patients available for follow-up *Recurrence rate*: 14.4%
Khan and Hayat^19^	18 patients in the surgical excision group (15 females, 3 males); average age: 31 y (range: 17-45)	Compressive dressing applied (length of time not specified)	*Average follow-up duration*: 1 y *Recurrence rate*: 5.6%
Kuliński et al^20^	198 patients	Immobilization in a short plaster splint (as distal as the metacarpophalangeal joints) was used for 5-7 d. Then, a rehabilitation program was started with a gradually increase in range of wrist joint motion	*Average follow-up duration*: 38 mo *Recurrence rate*: 24 (12.1%) patients *Pain*: less than or equal to 3 (assessed with VAS) *Grip strength*: no cases of weakening postoperation *Symptom resolution*: all resolved within 6 mo postoperation *Wound healing complications*: swelling and redness in 10 patients, purulent infection in 2 patients *Scarring*: widened, unaesthetic postoperative scar in 15 patients, keloid formation in 4 patients *ROM*: persistent limitation of palmar flexion of the wrist ranging from 10° to 15° in 6 patients
Lee et al^21^	20 patients (12 females, 8 males); average age: 34.4 y (range: 18-60).	Wrist was immobilized in a compressive dressing and volar splint for 5 d. Then, patients were permitted to mobilize the wrist and advised to avoid strenuous work for approximately 4 wk	*Average follow-up duration*: 32 mo *Pain*: 0.8 postoperatively (assessed with VAS). 85% of patients reported that their pain was much improved or very much improved. No patients’ pain worsened postoperatively. *Satisfaction*: 9.3 (assessed with VAS) *PRWE score*: 2.3 *Recurrence rate*: 3 (15%) patients. *Complications*: nil
Limpaphayom and Wilairatana^22^	11 patients in surgical excision group (9 females, 2 males); average age: 29.9 y	Compressive dressing (no length of time specified)	*Average follow-up duration*: 6 mo *Recurrence rate*: 18.2%
Öhman and Önne^23^	112 surgically treated DWGs	Plaster splint applied for 10 d	Results from 15-y follow-up period *Recurrence rate*: 12%

*Note.* DWG = dorsal wrist ganglion; ROM = range of motion; VAS = visual analog scale; PRWE = Patient-Rated Wrist Evaluation.

**Table 2.** Summary of Studies (Arthroscopic Dorsal Wrist Ganglion Excision).

Study	Population	Postoperative protocol	Postoperative outcomes
Ahsan and Yao^24^	27 patients (20 females, 7 males); average age: 33 y (range: 17-63)	Splint applied for 2 wk, after which normal range of motion exercises were recommended	*Average follow-up duration*: 25 mo. *Recurrence rate*: 3.7%
Aslani et al^25^	52 patients (40 females, 12 males); average age: 29.8 y	Bulky dressing applied (length of time not specified), patients encouraged to avoid strenuous activity for 6 wk	*Average follow-up duration*: 39.2 mo. *Recurrence rate*: 17.3% *ROM*: 12.7° and 10.5° improvement in flexion and extension, respectively *Grip strength*: 6.4 mmHg increase (measured with Jamar dynamometer) *Patient satisfaction*: 90.4% patients satisfied with treatment and results
Borisch^26^	40 patients (26 females, 14 males); average age: 29.3 y (range: 12-55); occult ganglions	Soft, well-padded dressing applied (length of time not specified), and patients allowed to mobilize wrist immediately; avoid strenuous exercise for 3 wk	*Average follow-up duration*: 36.2 mo; 30 patients available for follow-up *Pain at rest*: decreased from 2.7 to 0.3 (measured with Numeric rating scale) *Pain on load*: decreased from 7.4 to 2.3 (measured with Numeric rating scale) *ROM*: 13% reported residual limitations *Grip strength*: 13% reported reduced grip strength *Patient satisfaction*: 97% patients satisfied
Fernandes et al^27^	34 patients (25 females, 9 males); average age: 29.7 y (range: 11-53)	Short plaster splint was applied for 1 wk, after which a removable brace was advised for 2 wk	*Average follow-up duration*: 81 mo *QuickDASH score*: 2.3 points average *Pain*: 0.54 (assessed with VAS) *ROM*: full range of wrist movement was recovered by all patients *Mean palmar grip strength*: 29.4 kgf *Recurrence*: 1 case *Postoperative complications*: 1 case of hypertrophic scarring, no severe or moderate complications
Gallego and Mathoulin^28^	114 patients (87 females, 27 males); average age: 33.1 y (range: 12-63)	Bulky dressing applied (length of time not specified), and gentle hand movements encouraged in immediate postoperative period; no strenuous activity for several weeks	*Average follow-up duration*: 42.3 mo *Recurrence rate*: 12.3% (after an average of 16.86 mo) *Pain*: 100% patients with preoperative pain reported no pain postoperatively *Grip strength*: increase from 22.2 to 31.9 mmHg (*P* < .005) (measured with Jamar dynamometer) *ROM*: 15° increase in flexion, and 11.1° increase in extension after surgery (*P* = .001) *Time off work*: 11 d *Patient satisfaction*: 96.5%
Kang et al^29^	41 patients (28 females, 13 males); average age: 33.7 y (range: 19-59)	Splint applied for 1 wk; patients encouraged to move hand and wrist, but avoid strenuous activity for 4-6 wk	*Average follow-up duration*: 38.9 mo; 41 patients available for follow-up *Recurrence rate*: 9.7% (after 16 mo) *Pain*: decreased from 2.4 to 0.6 (*P* < .001) 2 y after surgery (assessed with VAS) *Grip strength*: increased from 28 kg of force to 36 kg of force (*P* < .001) 2 y after surgery (measured with dynamometer) *ROM*: no change from preoperative to 2 y after surgery *Mayo Wrist Score*: increased from 74 to 91 (*P* < .001)
Kim et al^30^	111 patients (54 females, 57 males); average age: 34 y (range: 9-72)	Splint applied for 7-14 d, after which activity allowed as tolerated, and avoidance of strenuous activity for 4 wk	*Average follow-up duration*: 32 mo *Recurrence rate*: 11% *Pain*: 23% reported residual pain after surgery
Langner et al^31^	26 patients with DWG; average age: 33 y (range: 18-57)	For patients with ganglion excision only, elastic bandage applied (length of time not specified) and early mobilization encouraged; for patients with ganglion excision and TFCC reattachment, sugar tong splint applied for 4 wk	*Average follow-up duration*: 1 y *Recurrence rate*: 15.4% *Pain at rest*: decreased from 2.3 to 0 (*P* = .02) after surgery (assessed with VAS) *Pain on load*: decreased from 6.5 to 2.5 (*P* < .01) after surgery (assessed with VAS) *ROM*: normal in 92.3% of patients *Disability*: decreased from 30 to 11 (*P* < .01) after surgery (assessed with DASH)
Lee et al^21^	25 patients (15 females, 10 males); average age: 34.6 y (range: 19-56)	Wrist was immobilized in a compressive dressing and volar splint for 5 d. Then, patients were permitted to mobilize the wrist and advised to avoid strenuous work for approximately 4 wk	*Average follow-up duration*: 32 mo *Pain*: 1.9 postoperatively (assessed with VAS). 72% of patients reported that their pain was much improved or very much improved. No patients’ pain worsened postoperatively. *Satisfaction*: 7.8 (assessed with VAS) *PRWE score*: 12.6 *Recurrence*: 4 (16%) patients. *Complications*: nil.
Luchetti et al^32^	34 patients (24 females, 10 males); average age: 29 y (range: 13-51)	No splint applied for most patients; a palmar splint was applied for 1 wk in patients treated for a recurrent ganglion after open surgery, requiring extensive arthrolysis	*Average follow-up duration*:16 mo *Recurrence rate*: 5.9% *Pain*: 8.9% of patients reported residual pain after surgery *In primary ganglia . . .* *Grip strength*: remained the same (22 kg) *ROM*: flexion decreased from 69° to 55°; extension decreased from 68° to 65° *Return to work*:16 d *In recurrent ganglia . . .* *Grip strength*: remained the same (22 kg) *ROM*: flexion increased from 55° to 60°; extension increased from 65° to 70° *Return to work*: 20 d
Nishikawa et al^33^	37 patients (24 females, 13 males); average age: 40 y (range: 14-84)	No splint applied, and rehabilitation started on the fourth postoperative day	*Average follow-up duration*: 20 mo *Recurrence rate*: 5.4% *Average recovery time*: 16 d
Rizzo et al^5^	41 patients (24 females, 17 males); average age: 29.8 y (range: 11-56)	Varied; patients treated early in the series had splint applied for 2-3 wk; most of the other patients had splint applied for 3-7 d, after which activity was allowed as tolerated, with avoidance of strenuous activity for 4-6 wk	*Average follow-up duration*: 47.8 mo *Recurrence rate*: 4.9% *Pain*: decreased from 5.2 to 1.2 (*P* = .001) after surgery *Grip strength*: increased from 15.3 to 22.3 kg (*P* = .01) after surgery *ROM*: increased from 50° to 59° (NS), and 42° to 55° (*P* = .03) for flexion and extension, respectively
Yamamoto et al^34^	16 patients with DWG	Soft dressing with no splint was applied for 7 d; movement of fingers and wrists encouraged starting on the second postoperative day, with avoidance of strenuous activity for 1 mo	*Average follow-up duration*:21 mo *Recurrence rate*: 12.5%

*Note.* DWG = dorsal wrist ganglion; TFCC = triangular fibrocartilage complex; NRS = numeric rating scale; QuickDASH = shortened disabilities of the arm, shoulder, and hand questionnaire; VAS = visual analog scale; PRWE = Patient-Rated Wrist Evaluation; ROM = range of motion.

Included studies had a high degree of bias as evaluated by the ROBINS-I and Cochrane RoB 2.0 tools (Table 3).^10,11^ Of the 21 nonrandomized studies, 9 studies had critical and 12 studies had serious RoB. The 3 RCTs had high levels of bias related to the outcomes measured. The interreliability kappa score was 0.99 for RoB assessment.

**Table 3.** Risk of Bias (RoB) Assessment (Risk of Bias in Non-Randomized Studies-of Interventions (ROBINS-I) and Cochrane RoB 2.0).

Study	Randomized process	Confounding	Selection of participants	Intervention classification	Deviations from intended intervention	Missing data	Outcome measurement	Selection of reported result	Overall
Ahsan and Yao^24^		Moderate	Moderate	Moderate	Low	Low	Serious	Low	Serious
Angelides and Wallace^12^		Moderate	Moderate	Low	Moderate	Moderate	Critical	Low	Critical
Aslani et al^25^		Serious	Moderate	Low	Moderate	Low	Critical	Low	Critical
Balazs et al^13^		Moderate	Low	Low	Low	Moderate	Serious	Low	Serious
Borisch^26^		Moderate	Moderate	Low	Low	Low	Critical	Low	Critical
Clay and Clement^14^		Critical	Moderate	Low	Moderate	Low	Serious	Low	Critical
Fernandes et al^27^		Serious	Moderate	Low	Low	Serious	Serious	Low	Serious
Gallego and Mathoulin^28^		Serious	Moderate	Low	Low	Low	Serious	Low	Serious
Gündeş et al^15^		Serious	Moderate	Low	Low	Low	Serious	Low	Serious
Hwang et al^16^		Moderate	Moderate	Moderate	Moderate	Moderate	Serious	Low	Serious
Jagers Op Akkerhuis et al^17^	Low				High	Low	High	Some concerns	High
Janzon and Niechajev^18^		Moderate	Low	Low	Low	Low	Serious	Low	Serious
Kang et al^29^		Serious	Moderate	Low	Low	Low	Serious	Low	Serious
Khan and Hayat^19^	Some concerns				High	Low	High	Some concerns	High
Kim et al^30^		Moderate	Low	Low	Moderate	Moderate	Serious	Low	Serious
Kuliński et al^20^		Serious	Moderate	Low	Moderate	Low	Serious	Low	Serious
Langner et al^31^		Critical	Serious	Low	Moderate	Moderate	Serious	Low	Critical
Lee et al^21^		Serious	Moderate	Low	Moderate	Low	Low	Low	Serious
Limpaphayom and Wilairatana^22^	Low				High	Low	High	Low	High
Luchetti et al^32^		Serious	Moderate	Low	Moderate	Low	Serious	Low	Serious
Nishikawa et al^33^		Serious	Moderate	Low	Moderate	Low	Critical	Low	Critical
Öhman and Önne^23^		Serious	Low	Low	Moderate	Low	Critical	Low	Critical
Rizzo et al^5^		Critical	Moderate	Low	Moderate	Low	Critical	Low	Critical
Yamamoto et al^34^		Critical	Moderate	Low	Moderate	Low	Serious	Low	Critical

#### Open DWG excision

Of the 12 studies on open DWG excision (Table 1), 6 (50%) used rigid immobilization of the wrist with splinting, 5 (42%) used soft dressings with variability in allowed wrist movement due to pressure and bulk, and 1 (8%) used either. The limited mobility time ranged from 48 hours to 2 weeks, with 3 (25%) studies not specifying the exact length of time.

The follow-up duration ranged from 6 months to 15 years. Angelides and Wallace^12^ reported the lowest ganglion recurrence rate of 0.9% after 9-month follow-up. The postoperative protocol involved a bulky bandage or plaster splint. However, the duration of the postoperative protocol was not specified. In contrast, Jagers Op Akkerhuis and colleagues^17^ reported the highest recurrence rate (28%) in patients immobilized with a pressure bandage for 48 hours after DWG excision. The follow-up duration was not specified.

#### Arthroscopic DWG excision

Among the 13 studies on arthroscopic DWG excision (Table 2), 6 (46%) used rigid immobilization, 5 (39%) allowed some wrist movement, and 2 (15%) studies did not limit wrist movement. Of the studies that limited wrist mobility, the limited mobility time ranged from 5 days to 3 weeks, with 4 (31%) studies not specifying the exact length of time.

The patient follow-up duration ranged from 12 to 81 months. Fernandes and colleagues^27^ presented the lowest recurrence rate of 2.9% after 81-month follow-up. In this study, a short plaster splint was applied for 1 week and followed by a removable brace for 2 weeks. The highest recurrence rate (17.3%) was reported by Aslani and colleagues^25^ after 39.2-month follow-up. These authors used a bulky dressing for an unspecified length of time.

#### Patient outcomes

In general, functional outcomes were inconsistently reported in all included studies. Therefore, comparisons to determine the relative effectiveness of the various immobilization protocols were not possible.

In open DWG excision, Clay and Clement^14^ postoperative protocol had the best outcomes for postoperative wrist range of motion. Patients’ wrists were immobilized in a plaster slab for 10 days postoperatively. “Normal” range of motion was restored for all 51 (100%) patients available for follow-up. Gündeş and colleagues^15^ showed postoperative wrist range of motion was most severely reduced in patients immobilized with a bulky compressive dressing for 2 weeks. Specifically, in patients with a bulky compressive dressing, 11.5% of patients had a 20° or more decrease in range of motion. Lee and colleagues^21^ reported the best improvement in pain, where 85% of patients reported that their pain was much improved or very much improved. These authors immobilized the wrist in a compressive dressing and volar splint for 5 days.

In terms of arthroscopic DWG excision, Fernandes and colleagues^27^ reported the best outcomes for postoperative range of motion, with all patients recovering full range of motion using the previously described postoperative protocol. Luchetti and colleagues^32^ reported a decrease in range of motion, with no splint applied postoperatively. Gallego and colleagues^28^ had the best documented improvement in pain, where 100% of patients who experienced preoperative pain reported no postoperative pain. These patients were immobilized with a bulky dressing for an unspecified length of time.^28^

Patient satisfaction was reported in 4 studies. Within the arthroscopic DWG excision group, when immobilized with a bulky dressing, high satisfaction rates were reported, ranging from 90.4% to 97%.^25,26,28^ However, Lee and colleagues^21^ performed both open and arthroscopic DWG excision, immobilizing their patients with a compressive dressing and volar splint. Within this study, patient satisfaction was reported to be better in the open group (9.3 vs 7.8 using visual analog scale [VAS], *P* = .042).^21^

Time to return to work was only reported in 2 studies, both of which were DWG that underwent arthroscopic excision.^28,32^ Patients immobilized with a bulky dressing for an unspecified period of time returned to work after 11 days, compared with 16 and 20 days in nonimmobilized patients with primary and recurrent ganglia, respectively.^28,32^

The lack of consistent outcome reporting made it impossible to make inferences about the effect of the immobilization protocols on grip strength.

### Survey of Canadian Hand Surgeons

Of the 65 Canadian hand surgeons contacted, 22 completed the online survey (34%). For surgeons who fully immobilized the wrist postoperatively (45%; n = 10/22), the majority do so for 8 to 14 days (80%; n = 8/10). The most common dressing applied is a volar wrist splint (80%; n = 8/10) in 30° of extension or less (60%; n = 6/10). The most cited purpose of postoperative immobilization is for improving patient comfort (50%; n = 5/10). There were 2 exceptions to immobilization expressed in this surgeon group, including not splinting patients older than 70 years and not splinting patients with specific phobias related to wrist immobilization.

For the hand surgeons who did not immobilize the wrist postoperatively (55%; n = 12/22), most applied a soft dressing allowing full mobility (75%; n = 9/12), while others used a compression dressing (17%; n = 2/12), or a soft dressing permitting a limited range of motion of wrist (8%; n = 1/12). One surgeon noted that immobilization may be necessary should an incision and repair of the extensor retinaculum be required.

## Discussion

The results of the systematic review highlight the lack of consensus amongst hand surgeons regarding the optimal postoperative care protocol in DWG excision patients. Although many of the included studies did employ some form of either rigid or limited immobilization, the type of dressing and the length of time for which it is applied are highly variable. Our survey of Canadian hand surgeons corroborates the highly variable practices of immobilization of the wrist after surgical excision of DWG. Interestingly, surveyed surgeons who did immobilize the wrist postoperatively most often limited the duration to 1 to 2 weeks, but never for more than 3 weeks. In contrast, many surgeons found no need for immobilization. The variability of postoperative immobilization has been documented in other areas of hand surgery, including carpal tunnel release.^35^ Despite critical evaluation through 5 RCTs concluding that splinting following carpal tunnel release does not have an appreciable effect on postoperative outcomes, surgeons’ opinions and practices remain divided. As with other aspects of surgical practice, deciding upon a specific postoperative dressing can be influenced by personal experience or learned behaviors from mentors.

The resources necessary for a RCT is likely not indicated for our clinical question as both groups seemingly have overall very good outcomes but a core outcome set would allow even work done as a case series to potentially meaningfully answer this question through meta-analysis. There is inconsistent reporting of outcomes in the DWG literature. For example, range of motion, grip strength, and pain outcomes were reported as either a percentage of patients experiencing improvement, a percentage of patients with residual symptoms, or as a preoperative and postoperative score on a questionnaire or measuring tool. The variation in outcome reporting makes it difficult to amalgamate these results into a meta-analysis. As a result, although preliminary inferences can be made about the relative success of the immobilization protocols, the degree to which 1 protocol is better or worse than the other remains unclear, especially in the context of varying surgical techniques. This substantiates the need for standardized core outcomes, such as those being developed by the Core Outcomes in Effectiveness Trials initiative.^36^

There are several limitations to our study. First, it is acknowledged that functional and patient-reported outcomes are influenced by not only postoperative care as we have studied but also surgical technique. Second, many included studies were observational in nature, with only 3 RCTs. Third, our survey results may be prone to selection bias.

## Conclusion

This systematic review of the literature has revealed a paucity of evidence available to inform the decision of wrist immobilization following DWG excision, either open or arthroscopic. Similarly, a survey of Canadian hand surgeons demonstrates current practice variability in postoperative dressings. Together, the data presented suggest that limited immobilization of 2 weeks or less or no immobilization after surgery does not meaningfully affect patient outcome after surgery. To detect a difference, the cumulative power of primary studies that employ a similar set of core outcomes that include patient-reported outcomes is necessary.

## Supplemental Material

sj-pdf-1-han-10.1177_15589447211014631 – Supplemental material for Immobilization of the Wrist After Dorsal Wrist Ganglion Excision: A Systematic Review and Survey of Current Practice Click here for additional data file. Supplemental material, sj-pdf-1-han-10.1177_15589447211014631 for Immobilization of the Wrist After Dorsal Wrist Ganglion Excision: A Systematic Review and Survey of Current Practice by Chloe R. Wong, Marta Karpinski, Alexandra C. Hatchell, Mark H. McRae, Jessica Murphy and Matthew C. McRae in HAND

## References

[1] Meena S Gupta A. Dorsal wrist ganglion: current review of literature. J Clin Orthop Trauma. 2014;5(2):59-64. 25983472 10.1016/j.jcot.2014.01.006 PMC4085360

[2] *Imaging of Soft Tissue Tumors* . New York, NY: Springer-Verlag; 2001.

[3] Doyle RW. Ganglia and superficial tumours. Practitioner. 1946;156:267-277. 21019429

[4] Psaila JV Mansel RE. The surface ultrastructure of ganglia. J Bone Joint Surg Br. 1978;60-B(2):228-233. 659471 10.1302/0301-620X.60B2.659471

[5] Rizzo M Berger RA Steinmann SP , et al. Arthroscopic resection in the management of dorsal wrist ganglions: results with a minimum 2-year follow-up period. J Hand Surg Am. 2004;29(1):59-62. 14751105 10.1016/j.jhsa.2003.10.018

[6] Osterman AL Raphael J. Arthroscopic resection of dorsal ganglion of the wrist. Hand Clin. 1995;11(1):7-12. 7751333

[7] Dias JJ Dhukaram V Kumar P. The natural history of untreated dorsal wrist ganglia and patient reported outcome 6 years after intervention. J Hand Surg Eur Vol. 2007;32(5):502-508. 17950209 10.1016/J.JHSE.2007.05.007

[8] Dias J Buch K. Palmar wrist ganglion: does intervention improve outcome? A prospective study of the natural history and patient-reported treatment outcomes. J Hand Surg Br. 2003;28(2):172-176. 12631492 10.1016/s0266-7681(02)00365-0

[9] Craik JD Walsh SP. Patient outcomes following wrist ganglion excision surgery. J Hand Surg Eur Vol. 2012;37(7):673-677. 22223582 10.1177/1753193411434376

[10] Sterne JAC Hernán MA Reeves BC , et al. ROBINS-I: a tool for assessing risk of bias in non-randomised studies of interventions. BMJ. 2016;355:i4919. 27733354 10.1136/bmj.i4919 PMC5062054

[11] Sterne JAC Savović J Page MJ , et al. RoB 2: a revised tool for assessing risk of bias in randomised trials. BMJ. 2019;366:l4898. 31462531 10.1136/bmj.l4898

[12] Angelides AC Wallace PF. The dorsal ganglion of the wrist: its pathogenesis, gross and microscopic anatomy, and surgical treatment. J Hand Surg Am. 1976;1(3):228-235. 1018091 10.1016/s0363-5023(76)80042-1

[13] Balazs GC Donohue MA Drake ML , et al. Outcomes of open dorsal wrist ganglion excision in active-duty military personnel. J Hand Surg Am. 2015;40(9):1739-1747. 26228481 10.1016/j.jhsa.2015.05.030

[14] Clay NR Clement DA. The treatment of dorsal wrist ganglia by radical excision. J Hand Surg Br. 1988;13(2):187-191. 3385297 10.1016/0266-7681_88_90135-0

[15] Gündeş H Cirpici Y Sarlak A , et al. Prognosis of wrist ganglion operations. Acta Orthop Belg. 2000;66(4):363-367. 11103488

[16] Hwang JJ Goldfarb CA Gelberman RH , et al. The effect of dorsal carpal ganglion excision on the scaphoid shift test. J Hand Surg Br. 1999;24(1):106-108. 10190618 10.1016/s0266-7681(99)90053-0

[17] Jagers Op Akkerhuis M Van Der Heijden M Brink PR. Hyaluronidase versus surgical excision of ganglia: a prospective, randomized clinical trial. J Hand Surg Br. 2002;27(3):256-258. 12074614 10.1054/jhsb.2002.0764

[18] Janzon L Niechajev IA. Wrist ganglia: incidence and recurrence rate after operation. Scand J Plast Reconstr Surg. 1981;15(1):53-56. 7268314 10.3109/02844318109103412

[19] Khan PS Hayat H. Surgical excision versus aspiration combined with intralesional triamcinolone acetonide injection plus wrist immobilization therapy in the treatment of dorsal wrist ganglion; a randomized controlled trial. J Hand Microsurg. 2011;3(2):55-57. 23204769 10.1007/s12593-011-0039-6 PMC3172357

[20] Kuliński S Gutkowska O Mizia S , et al. Dorsal and volar wrist ganglions: the results of surgical treatment. Adv Clin Exp Med. 2019;28(1):95-102. 30070079 10.17219/acem/81202

[21] Lee HLL Lee KH Koh KH , et al. Excision of painful dorsal wrist ganglion by open or arthroscopic approach: a comparison study. Acta Orthop Belg. 2017;83(2):315-321. 30399997

[22] Limpaphayom N Wilairatana V. Randomized controlled trial between surgery and aspiration combined with methylprednisolone acetate injection plus wrist immobilization in the treatment of dorsal carpal ganglion. J Med Assoc Thai. 2004;87(12):1513-1517. 15822550

[23] Ohman U Onne L. Carpal ganglia. A follow-up study. Scand J Plast Reconstr Surg. 1971;5(2):110-115. 5136051 10.3109/02844317109042949

[24] Ahsan ZS Yao J. Arthroscopic dorsal wrist ganglion excision with color-aided visualization of the stalk: minimum 1-year follow-up. Hand (N Y). 2014;9(2):205-208. 24839422 10.1007/s11552-013-9570-1 PMC4022959

[25] Aslani H Najafi A Zaaferani Z. Prospective outcomes of arthroscopic treatment of dorsal wrist ganglia. Orthopedics. 2012;35(3):e365-e370. 22385448 10.3928/01477447-20120222-13

[26] Borisch N. Arthroscopic resection of occult dorsal wrist ganglia. Arch Orthop Trauma Surg. 2016;136(10):1473-1480. 27535671 10.1007/s00402-016-2539-0

[27] Fernandes CH Meirelles LM Raduan Neto J , et al. Arthroscopic resection of dorsal wrist ganglion: results and rate of recurrence over a minimum follow-up of 4 years. Hand (N Y). 2019;14(2):236-241. 29185350 10.1177/1558944717743601 PMC6436138

[28] Gallego S Mathoulin C. Arthroscopic resection of dorsal wrist ganglia: 114 cases with minimum follow-up of 2 years. Arthroscopy. 2010;26(12):1675-1682. 20952152 10.1016/j.arthro.2010.05.008

[29] Kang HJ Koh IH Kim JS , et al. Coexisting intraarticular disorders are unrelated to outcomes after arthroscopic resection of dorsal wrist ganglions. Clin Orthop Relat Res. 2013;471(7):2212-2218. 23430724 10.1007/s11999-013-2870-5 PMC3676617

[30] Kim JP Seo JB Park HG , et al. Arthroscopic excision of dorsal wrist ganglion: factors related to recurrence and postoperative residual pain. Arthroscopy. 2013;29(6):1019-1024. 23726108 10.1016/j.arthro.2013.04.002

[31] Langner I Krueger PC Merk HR , et al. Ganglions of the wrist and associated triangular fibrocartilage lesions: a prospective study in arthroscopically-treated patients. J Hand Surg Am. 2012;37(8):1561-1567. 22749480 10.1016/j.jhsa.2012.04.042

[32] Luchetti R Badia A Alfarano M , et al. Arthroscopic resection of dorsal wrist ganglia and treatment of recurrences. J Hand Surg Br. 2000;25(1):38-40. 10763721 10.1054/jhsb.1999.0290

[33] Nishikawa S Toh S Miura H , et al. Arthroscopic diagnosis and treatment of dorsal wrist ganglion. J Hand Surg Br. 2001;26(6):547-549. 11884110 10.1054/jhsb.2001.0620

[34] Yamamoto M Kurimoto S Okui N , et al. Sonography-guided arthroscopy for wrist ganglion. J Hand Surg Am. 2012;37(7):1411-1415. 22633231 10.1016/j.jhsa.2012.04.012

[35] Isaac SM Okoro T Danial I , et al. Does wrist immobilization following open carpal tunnel release improve functional outcome? A literature review. Curr Rev Musculoskelet Med. 2010;3(1-4):11-17. 21063494 10.1007/s12178-010-9060-9 PMC2941580

[36] “Core Outcome Measures in Effectiveness Trials.” COMET Initiative | Home, www.comet-initiative.org/.

