# Supplementary material for: Immobilization of the Wrist After Dorsal Wrist Ganglion Excision: A Systematic Review and Survey of Current Practice
Source: Hand (N Y). 2021 Jun 6;18(2):254–63. doi: 10.1177/15589447211014631 (PMC10035098; doi:10.1177/15589447211014631)
Supplement: sj-pdf-1-han-10.1177_15589447211014631 – Supplemental material for Immobilization of the Wrist After Dorsal Wrist Ganglion Excision: A Systematic Review and Survey of Current Practice [file sj-pdf-1-han-10.1177_15589447211014631.pdf]

CINAHL Search Strategy

S1. (MH "Ganglion Cysts") OR (MH "Cysts") OR (MH “Synovial Cyst”)
S2. TI ((ganglion or synovial) N1 cyst*) or AB ((ganglion or synovial) N1 cyst*)
S3. TI "bible bump" OR AB "bible bump*"
S4. TI "gideon's disease" OR AB "gideon's disease"
S5. TI "olamide* cyst*" OR AB "olamide* cyst*"
S6. TI bible cyst* OR AB bible cyst*
S7. TI ((intraosseous or intra- osseous) N1 (cyst* or gangli*)) or AB ((intraosseous or intra-osseous) N1 (cyst* or gangli*))
S8. (MH "Bone Cysts") OR (MH "Bone Cysts, Aneurysmal")
S9. TI (cysts or cyst) OR AB (cysts or cyst)
S10. S1 OR S2 OR S3 OR S4 OR S5 OR S6 OR S7 OR S8 OR S9
S11. (MH "Wrist")
S12. (MH "Wrist Joint")
S13. (MH "Carpal Joints")
S14. TI (wrist* or carpal*) OR AB (wrist* or carpal*)
S15. S11 OR S12 OR S13 OR S14
S16. TI ((wrist* or carpal*) N1 gangli*) OR AB ((wrist* or carpal*) N1 gangli*)
S17. S10 AND S15
S18. S16 OR S17

Embase Search Strategy

1. ganglion cyst/ or intraosseous ganglion/ or wrist ganglion/
2. synovial cyst/
3. cyst/
4. (cyst or cysts).ti,ab,kw.
5. bible bump*.ti,ab,kw.
6. Gideon's disease.ti,ab,kw.
7. bone cyst/ or aneurysmal bone cyst/
8. ((intraosseous or intra-osseous) adj1 gangli*).ti,ab,kw.
9. or/1-8
10. wrist/
11. triangular fibrocartilage/
12. carpal joint/
13. (wrist* or carpal*).ti,ab,kw.
14. or/10-13
15. 9 and 14
16. ((wrist* or carpal*) adj1 gangli*).ti,ab,kw.
17. 15 or 16
18. general surgery/
19. plastic surgery/
20. surgical technique/
21. surgery/
22. (surg* or operative or operation or excision or postoperative or postoperative).ti,ab,kw.
23. excision/
24. excision*.ti,ab,kw.
25. ganglionectomy/
26. (ganglionectom* or gangliectom*).ti,ab,kw.
27. su.fs.
28. postoperative care/ or postoperative period/
29. or/18-28
30. 17 and 29
31. external fixator/
32. splint/
33. (external fixator* or splint*).ti,ab,kw.
34. or/31-33
35. 17 and 34
36. 30 or 35
37. 17 and 29 and 34
38. remove duplicates from 36

Medline Search Strategy

1. Ganglion Cysts/
2. Synovial Cyst/
3. Cysts/
4. (cyst or cysts).ti,ab,kf.
5. bible bump*.ti,ab,kf.
6. Gideon's disease.ti,ab,kf.
7. bone cysts/ or bone cysts, aneurysmal/
8. ((intraosseous or intra-osseous) adj1 gangli*).ti,ab,kf.
9. 1 or 2 or 3 or 4 or 5 or 6 or 7 or 8
10. Wrist/
11. wrist joint/ or triangular fibrocartilage/
12. Carpal Joints/
13. (wrist* or carpal*).ti,ab,kf.
14. or/10-13
15. 9 and 14
16. ((wrist* or carpal*) adj1 gangli*).ti,ab,kf.
17. 15 or 16
18. General Surgery/
19. Surgery, Plastic/
20. Surgical Procedures, Operative/
21. (surg* or operative or operation or excision or postoperative or post- operative).ti,ab,kf.
22. excision*.ti,ab,kf.
23. Ganglionectomy/
24. (ganglionectom* or gangliectom*).ti,ab,kf.
25. su.fs.
26. Postoperative Care/ or Postoperative Period/
27. or/18-26
28. 17 and 27
29. external fixators/ or splints/
30. (external fixator* or splint*).ti,ab,kf.
31. 29 or 30
32. 17 and 31
33. 28 or 32
34. 27 and 31
35. 17 and 34
36. remove duplicates from 33

SportDiscus Search Strategy

S1. DE "CYSTS (Pathology)" OR DE “BONE cysts”
S2. TI (ganglion or synovial) N1 cyst* OR AB (ganglion or synovial) N1 cyst*
S3. TI (bible bump*) OR AB (bible bump*)
S4. TI (gideon's disease) or AB (gideon's disease)
S5. TI (olamide* cyst*) or AB (olamide* cyst*)
S6. (TI (bible cyst*) OR AB (bible cyst*))
S7. (TI (intraosseous or intra- osseious) N1 (cyst* or gangli*) OR AB (intraosseous or intra-osseous) N1 (cyst* or gangli*))
S8. cyst or cysts
S9. (S1 OR S2 OR S3 OR S4 OR S5 OR S6 OR S7 OR S8)
S10. DE "WRIST"
S11. TI (wrist* or carpal*) OR AB (wrist* or carpal*)
S12. S10 OR S11
S13. (S9 AND S12)
S14. TI ((wrist* or carpal*) N1 gangli*) OR AB ((wrist*or carpal*) N1 gangli*)
S15. (S13 OR S14)
